# Usefulness and limitation of indocyanine green fluorescence for detection of peritoneal recurrence after hepatectomy for hepatocellular carcinoma: a case report

**DOI:** 10.1186/s12893-021-01111-8

**Published:** 2021-03-02

**Authors:** Hikaru Hayashi, Akira Shimizu, Hiroaki Motoyama, Koji Kubota, Tsuyoshi Notake, Shinsuke Sugenoya, Kiyotaka Hosoda, Koya Yasukawa, Ryoichiro Kobayashi, Yuji Soejima

**Affiliations:** Division of Gastroenterological, Hepato-Biliary-Pancreatic, Transplantation and Pediatric Surgery, Department of Surgery, Shinshu University School of Medicine, 3-1-1 Asahi, Matsumoto, Nagano 390-8621 Japan

**Keywords:** Hepatocellular carcinoma, Peritoneal dissemination, Indocyanine green fluorescence

## Abstract

**Background:**

Peritoneal recurrence of hepatocellular carcinoma (HCC) after hepatectomy occurs rarely, accounting for less than 1% of all recurrences. Reported causes of such dissemination include a history of rupture of the original HCC, needle biopsy or puncture treatment, and surgical procedures. There is no consensus on the optimal treatment strategy for peritoneal dissemination. There have been few reports on assisting resection of peritoneal dissemination by using indocyanine green (ICG) fluorescence.

**Case presentation:**

A 57-year-old man underwent posterior sectionectomy for HCC. Six months later, computed tomography revealed multiple nodules suspected of indicating peritoneal dissemination. Various preoperative imaging studies demonstrated only four nodules, the doubling time of the tumors being rapid at 22 days. The nodules were therefore resected. ICG (0.5 mg/kg) was injected intravenously 2 days before the procedure, enabling identification of the nodules by their brightness in the operative field under near-infrared lighting. A total of eight lesions were detected during the procedure and resected, some of which had not been identified by preoperative imaging studies. We diagnosed peritoneal dissemination of HCC based on the pathological findings and their similarity to those of the original HCC. We concluded that the recurrences were likely attributable to exposure of the tumor to the serosa at the time of the original operation.

**Conclusions:**

Although ICG fluorescence is useful for identifying peritoneal dissemination of HCC, attention should be paid to the difficulty in detecting deep lesions and occurrence of false positives.

## Background

Although hepatocellular carcinoma (HCC) is the most common primary malignant tumor of the liver, peritoneal recurrence after hepatectomy is rare, accounting for less than 1% of all recurrences [1]. Reported causes of such dissemination include a history of ruptured HCC [2], needle biopsy or puncture treatment [3], and surgical procedures. In general, peritoneal dissemination of HCC has a poor prognosis and there is no consensus on the optimal treatment strategy. There have been few reports on assisting resection of peritoneal dissemination by using indocyanine green (ICG) fluorescence [4, 5]. We herein report using ICG fluorescence to assist resection of HCC peritoneal dissemination after hepatectomy.

## Case presentation

**Fig. 1:**
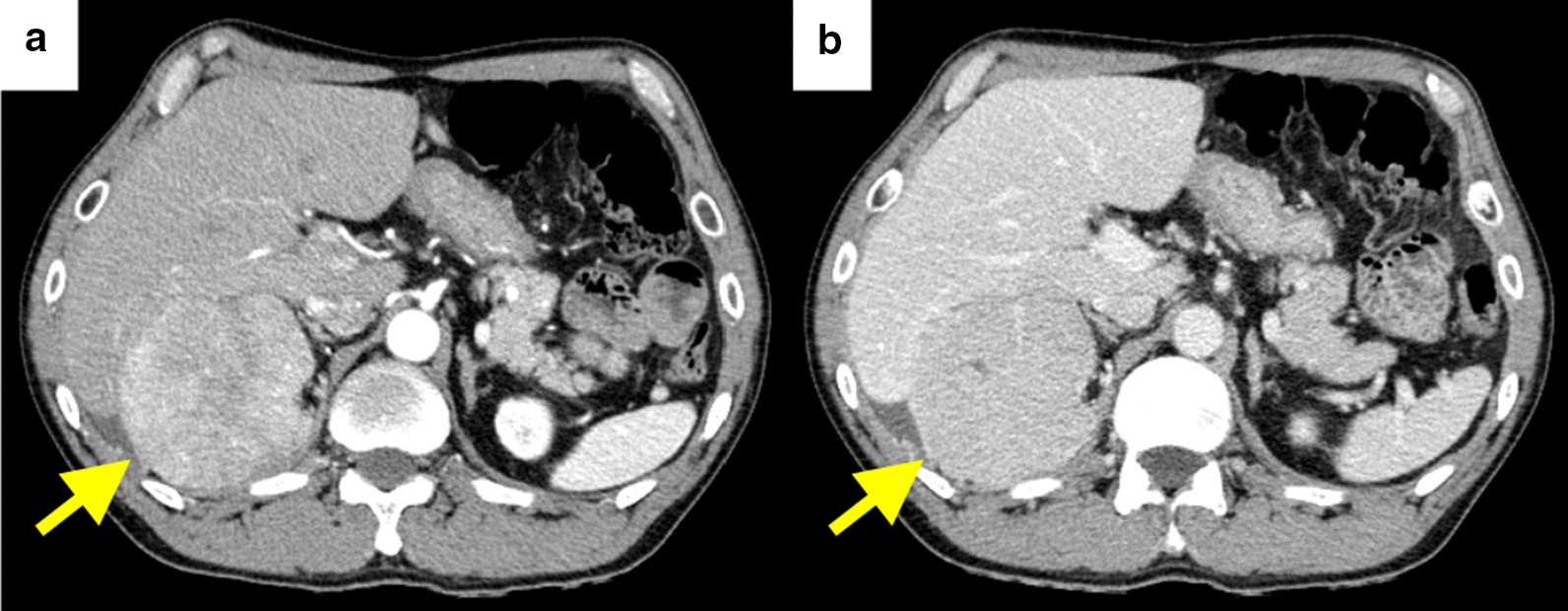
Contrast-enhanced abdominal computed tomography images showing a 10 cm diameter tumor protruding from segment 6 into the retroperitoneum. **a** Arterial phase. **b** Portal phase

**Fig. 2:**
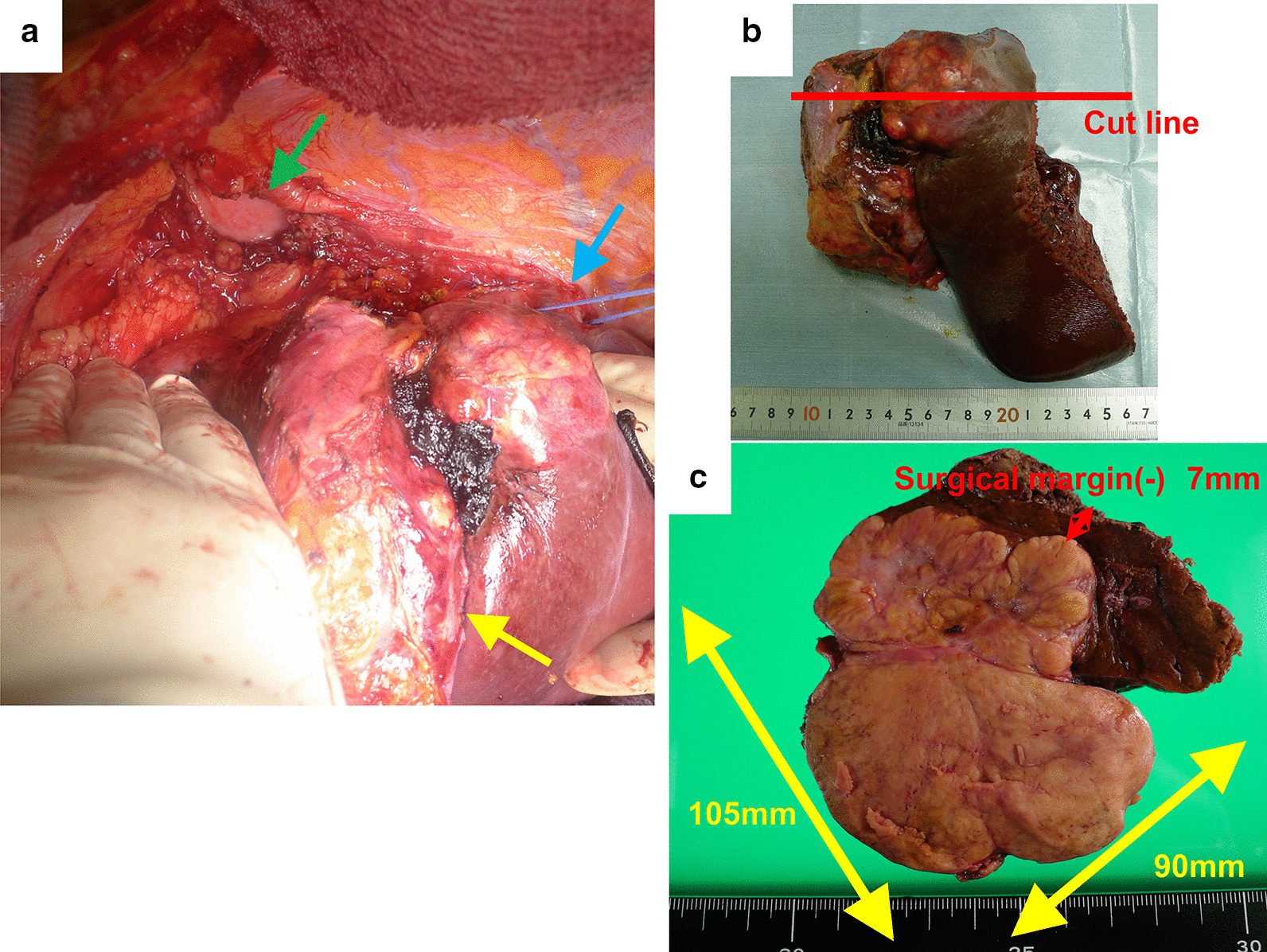
Intraoperative photograph and photograph of the resected specimen. **a** Intraoperative photograph. Yellow arrow, tumor; blue arrow, right hepatic vein; green arrow, partial resection of diaphragm. **b** The resected specimen. **c** Cut surface of the tumor. Exposure of the tumor to the serosa was confirmed

A 57-year-old man underwent posterior sectionectomy for a hepatic tumor of maximum diameter 10 cm that protruded from segment 6 into the retroperitoneum (Figs. 1 and 2). Histological examination of the primary tumor showed a simple nodular lesion with extranodular growth, and moderately to poorly differentiated HCC. The tumor had formed a capsule. Infiltration of the capsule and portal vein invasion (vp1) were noted. No intrahepatic or regional lymph node metastasis was observed. Surgical margins were negative. The pathological stage was III according to the staging system of the Liver Cancer Study Group of Japan [6].

**Fig. 3:**
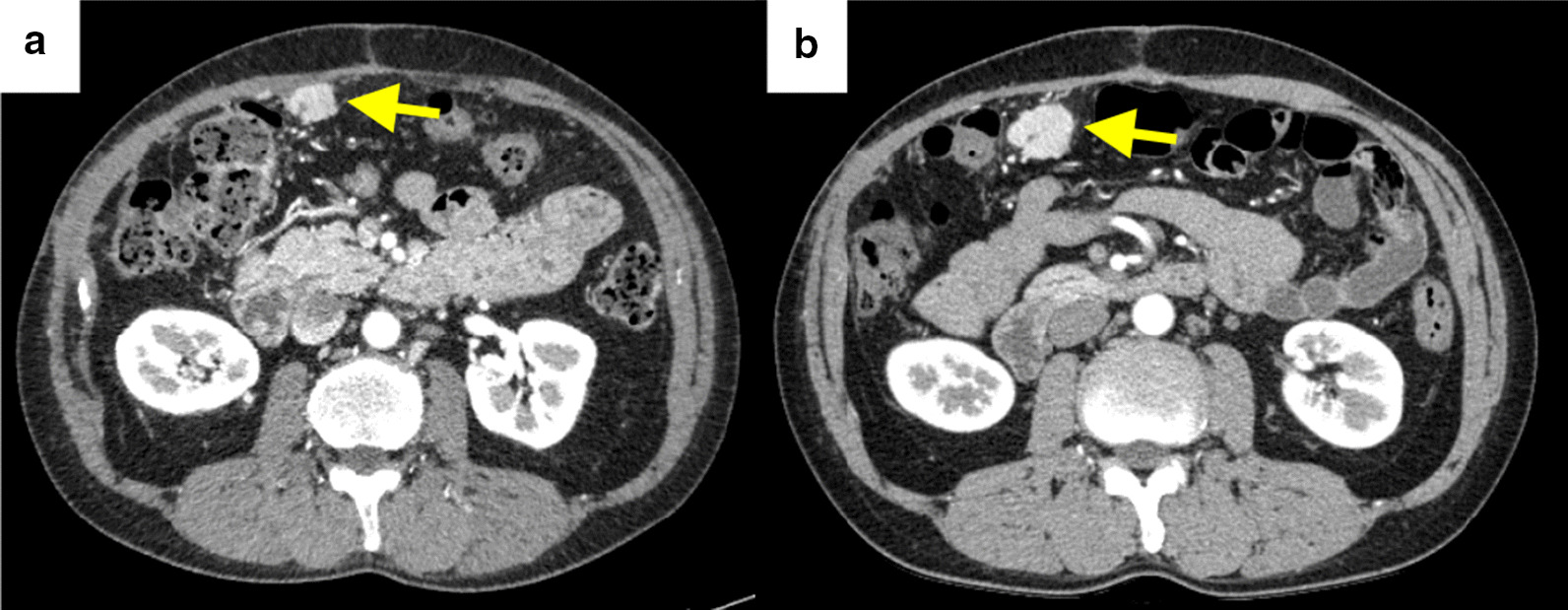
Contrast-enhanced abdominal computed tomography images **a **Preoperative image showing multiple nodules suspected of denoting peritoneal dissemination. Yellow arrow, biggest nodule, which had a doubling time of 22 days. **b** Image obtained 3 weeks after that shown in (**a**)

After 6 months with watchful waiting, follow-up computed tomography (CT) revealed multiple nodules suspected of indicating peritoneal dissemination (Fig. 3). The patient had dyslipidemia but no liver disease, and was taking no medication. The liver was impalpable, and there was no obvious abdominal tenderness or rebound pain. Laboratory data showed a white blood cell count of 2.75 × 10^9^ /L with normal differential counts and a C-reactive protein concentration of 0.5 mg/L. Concentrations of hepatobiliary enzymes were within normal limits, whereas those of alpha-fetoprotein (AFP) and protein induced by vitamin K absence or antagonist-II (PIVKA-II) were high at 1,480 ng/mL and 970 mAU/mL, respectively; these had tended to increase since the original operation. CT and magnetic resonance imaging (MRI) showed only four nodules in the abdominal cavity, the doubling times of which were rapid at 22 days. No other metastases were detected by these imaging studies.

**Fig. 4:**
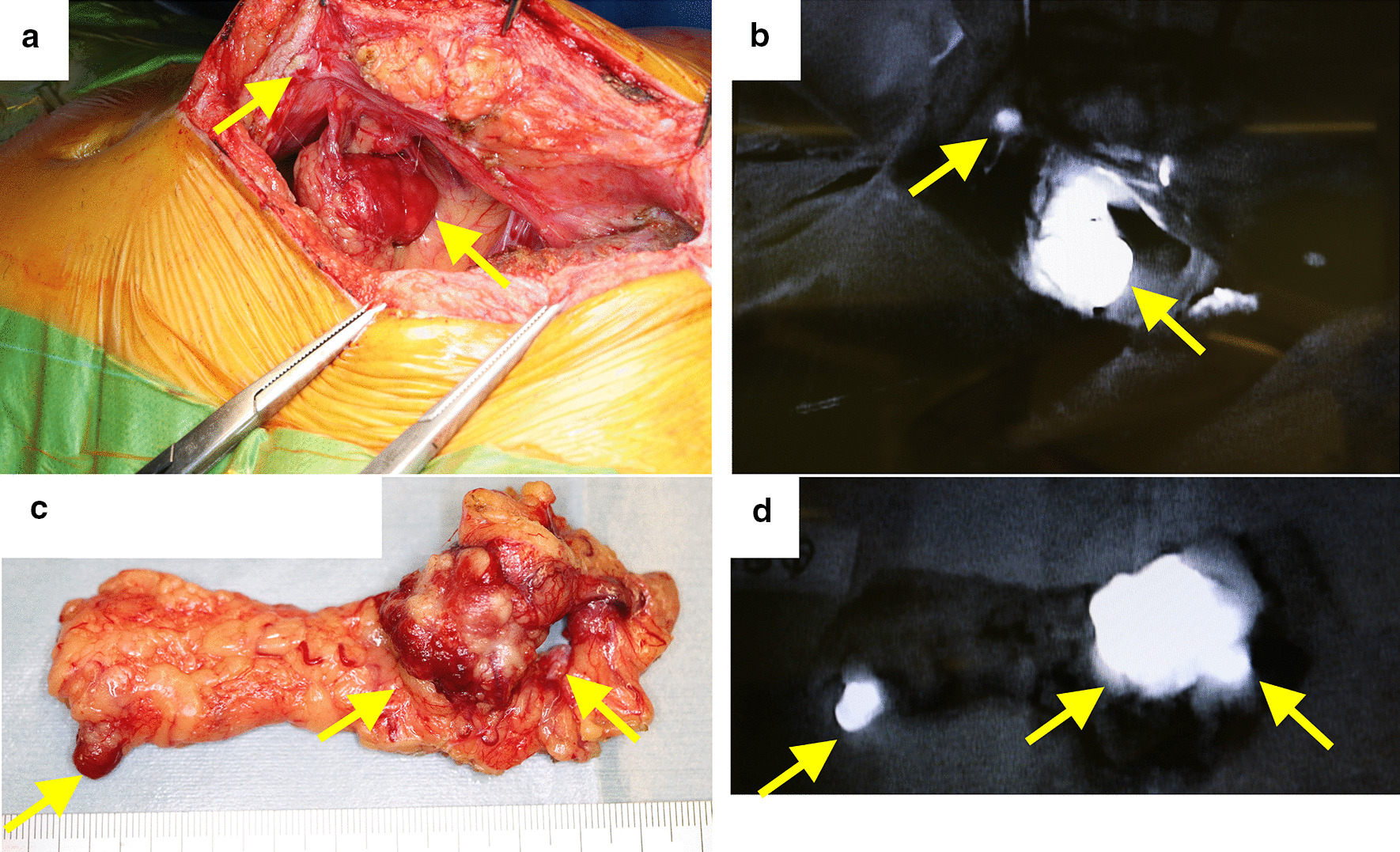
Intraoperative images and the resected specimen. **a** Intraoperative image taken with a normal camera. **b** Image obtained using a near-infrared light camera system. **c** Photograph of resected specimen image taken with a normal camera. **d** Photograph of resected specimen obtained using a near-infrared light camera system

**Fig. 5:**
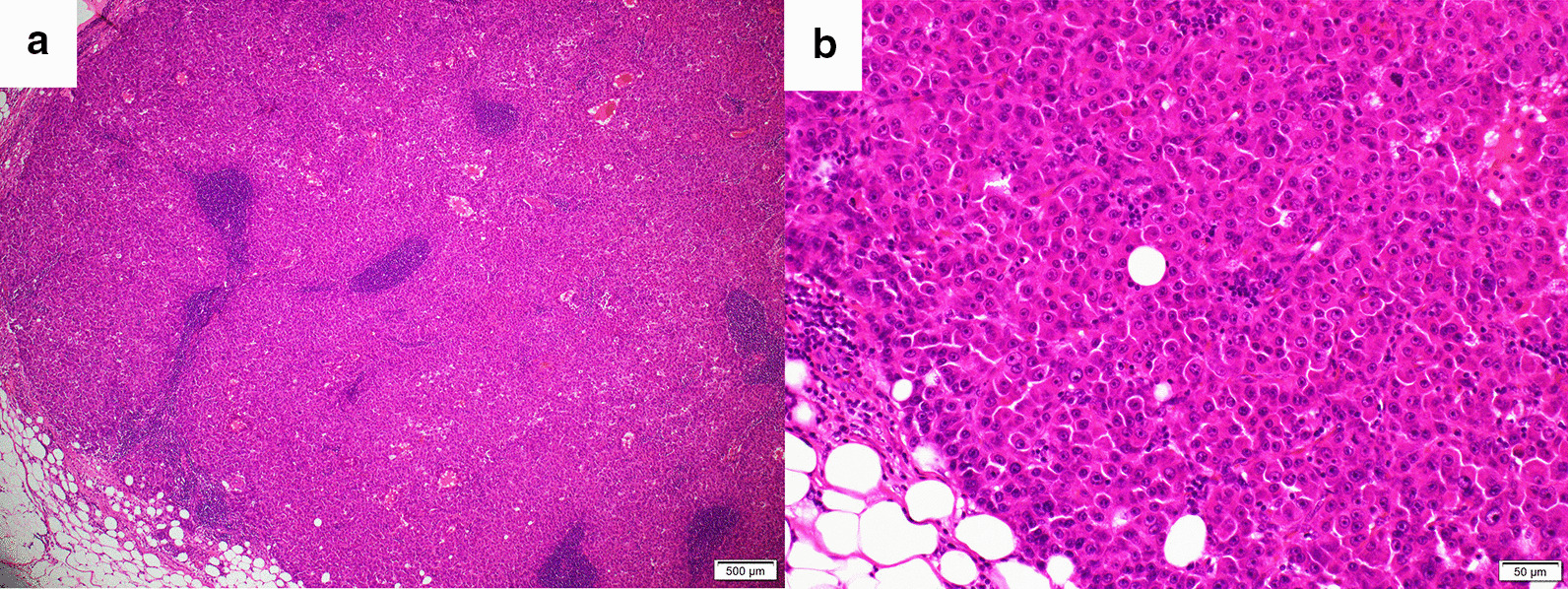
Photomicrographs showing proliferating tumor cells that have formed thick cord-like and pseudo glandular ductal structures and have invaded the fatty tissue beyond the serosa of the liver. The stain is hematoxylin and eosin. The surgical margin was negative. (**a**) Under low magnification (20x). (**b**) Under high magnification (200x)

On the basis of these findings, we diagnosed peritoneal dissemination of HCC after hepatectomy. Because of their rapid growth and apparent resectability, surgical resection was carried out. ICG (0.5 mg/kg) was injected intravenously 2 days before the procedure and the peritoneal nodules identified by their brightness in the operative field using an ICG near-infrared fluorescence imaging system (PDE™, Hamamatsu Photonics K.K., Hamamatsu, Japan). A total of eight lesions were thus identified and resected, including some that had not been identified by preoperative imaging studies (Fig. 4). However, there were no ICG signals on the surface of the remnant liver. The peritoneal cancer index (PCI) was 6 (right upper; the maximum tumor was 28 mm and the score 2, right flank; 14 mm and 2, epigastrium; 2 mm and 1, central; 2 mm and 1, respectively), and the completeness of cytoreduction (CC) score was 0. Histopathological examination of five of the eight resected lesions showed proliferating cancer cells, with some scattered small pseudo-glandular structures (Fig. 5). We diagnosed peritoneal dissemination of HCC based on the pathological findings and their similarity to those of the original HCC. We concluded that the recurrences were likely attributable to exposure of the tumor to the serosa at the time of the original operation. Additionally, three of the four resected lesions that had not been detected by preoperative imaging studies were found to consist of nonspecific fibrous tissue.

The postoperative course was smooth and the patient was discharged soon after surgery. Two months later, follow-up CT revealed further peritoneal dissemination that had not been detected by imaging studies performed preoperatively or intraoperative investigation. For these reasons, the patient was commenced on lenvatinib. Thereafter, the patient’s condition was stable and no new lesions were identified in the subsequent 10 months, during which there was a progressive decrease in concentrations of tumor markers.

## Discussion and conclusions

Peritoneal recurrence of HCC is rare. According to the report of the 20th nationwide follow-up survey of primary liver cancer in Japan [1], only 35 (0.5%) of 6468 registered patients had peritoneal recurrence. Nakashima et al. [7] reported finding metastases in the pouch of Douglas in 6.2% of 232 consecutive autopsy cases of HCC. In patients whose HCCs have not been ruptured, such metastases seem to occur only in the late stage of the disease because, as in gastric cancer [8], peritoneal dissemination involves a multi-step process that includes attachment of tumor cells to the peritoneal mesothelium, dissociation of mesothelial cells, invasion of the subperitoneal space, and proliferation with angiogenesis. Thus, the peritoneum may not be an optimal environment for development of HCC metastases. Another possible explanation for the discrepancy between the findings of the follow-up survey of primary liver cancer in Japan [1] and Nakashima et al.’s autopsy findings [7], may be the difficulty in making this diagnosis by ascitic fluid cytology or diagnostic imaging. Some established causes of peritoneal dissemination include a history of rupture of the original HCC [2], a history of needle biopsy or puncture treatment [3], and surgical procedures. Furthermore, Kow et al. [9] have reported that tumor diameter > 50 mm, presence of microvascular invasion, bile duct invasion, and positive resection margins are significant risk factors for peritoneal recurrence after hepatectomy. Our patient had no history of needle biopsy or puncture and no serosal damage had been identified during the original surgery. However, exposure of the tumor to the serosa was confirmed by histopathological examination of the original operative specimen; this was considered to be the main cause of the recurrences.

The treatment strategy for recurrences after hepatectomy is basically the same as the initial strategy; however, no consensus on treatment of peritoneal dissemination has yet been established. Although peritoneal dissemination of other gastrointestinal cancers is generally not resectable because of its frequent association with ascites, peritoneal dissemination of HCC characteristically takes the form of isolated or localized nodular lesion. Various treatments, including intraperitoneal hyperthermia or chemotherapy [10], systemic chemotherapy [11], and surgical resection have been tried, despite which the prognosis is generally extremely poor. However, some reported patients have achieved long term survival after dissection [12, 13]. Establishment of criteria for selection of patients who are candidates for surgery is important. Sugarbaker et al. [14] have reported that the prognosis is related to the sizes of the peritoneal tumors, extent of involvement, degree of tumor differentiation, and the presence or absence of residual cancer. These authors particularly emphasized the importance of the last factor. Therefore, the ability to completely resect peritoneal nodules has the greatest impact. Furthermore, Iida et al. [15] reported 92 cases of surgical resection of peritoneal metastases from HCC in Japan. These patients were classified using PCI and CC scores to determine whether peritoneal metastasectomy contributed to survival. Multivariate analysis revealed that a PCI score of ≤ 6 and CC score of 0 were independent prognostic factors. In our patient, the PCI was 4 (right upper; maximum tumor diameter 28 mm and score 2, right flank; 14 mm and 2, respectively) and the CC score was 0. According to the above analysis and preoperative imaging studies, we considered that upfront surgery was justifiable despite the false negative lesions (the PCI score of ≤ 6). However, if there had been more lesions than expected and complete resection had proved difficult, it would have been necessary to abandon the surgery and consider induction systemic chemotherapy or to consider induction chemotherapy early after surgery. Furthermore, we should have considered initiating chemotherapy immediately after the operation because of the high risk of recurrence. Moreover, preoperative positron emission tomography using 18F-flurodeoxyglucose (18F-FDG-PET)/CT to search systemically may better determine whether complete tumor clearance is possible. Sugiyama et al. [16] reported that 18F-FDG-PET detects 83% of extrahepatic metastases larger than 10 mm in greatest diameter and 13% of smaller lesions. Additionally, Böhm et al. [17] reported a sensitivity and specificity for detecting extrahepatic lesions of 63% and 60%, respectively. Although there are some differences between reports, it is generally agreed that 18F-FDG-PET/CT is a useful tool for detecting peritoneal metastases above a certain size. In retrospect, we should have performed an 18F-FDG-PET/CT scan on our patient considering how rapidly peritoneal recurrence occurred.

Reliable means of intraoperative identification of extrahepatic lesions are needed to achieve complete tumor clearance. The usefulness of ICG fluorescence in surgery has been widely discussed, including in the hepatobiliary-pancreatic fields, in which identification of tumors during laparoscopic hepatic resection [18–20], identification of biliary tract migration in laparoscopic cholecystectomy [21], and identification of the perfusion basin for gallbladder cancer [22] have been reported. To the best of our knowledge, only two studies have reported the usefulness of ICG fluorescence navigation in managing peritoneal dissemination of HCC [4, 5]. The ICG fluorescence method makes use of the fact that ICG emits fluorescence under near-infrared excitation by binding with albumin in blood. Although ICG is taken up into HCC cells in the same way as into normal hepatocytes, it characteristically stays in the tumor cells longer because they are unable to excrete it via the biliary tract. ICG may be more useful for extrahepatic than primary lesions because of the lack of fluorescence in tissues adjacent to extrahepatic lesions; this results in more pronounced contrast than with primary lesions. Sato et al. [23] reported that ICG fluorescence under near-infrared light had a 100% positive predictive value with a sensitivity of 96% for 33 extrahepatic lesions. Indeed, all of our patient’s lesions that had been identified by preoperative imaging studies were visualized as points of high brightness intraoperatively. Furthermore, the fluorescence enabled detection of lesions that had not been identified by preoperative imaging studies. We therefore consider that the ICG fluorescence method facilitates achieving no residual tumor status.

How effectively lesions can be detected using ICG fluorescence, however, depends on their depth: fluorescence is not visible when a lesion is deeply located. Several studies have reported that tumors located more than about 5–10 mm from the organ surface are not visualized by this method [19, 24, 25]. Moreover, Morita et al. [26] reported that fluorescence-negative hepatic tumors are significantly smaller than those that are positive. In our patient, recurrence was detected soon after surgery, pointing to a limitation in using the ICG fluorescence method to detect extrahepatic lesions. In addition, detection of ICG fluorescence in the peritoneum does not necessarily mean recurrence; several false positives occurred in our patient, the mechanism for this is unclear. According to one report, one of 64 hyperfluorescent nodules was found to be non-cancerous; no possible mechanisms were mentioned [27]. Although the majority of explanations for false positives include sutures or blood, if those possibilities are ruled out, one way of discriminating true positives may be that a spherical shape with a clear, bright, outline denotes a metastatic lesion.

We have herein reported using ICG fluorescence to facilitate resection of peritoneal metastases of HCC after hepatectomy, a procedure that has been reported infrequently. Although peritoneal dissemination of HCC has a poor prognosis, selected patients may benefit from complete excision of peritoneal lesions. Assisting surgery by intraoperative ICG fluorescence may be useful in preventing residual tumors. However, it should be noted that all metastatic lesions may not be detected and false positives can occur.

## Data Availability

The dataset supporting the conclusions of this article is included within the article.

## Abbreviations

18F-FDG-PET: Positron emission tomography using 18F-flurodeoxyglucose
AFP: Alpha-fetoprotein
CC: Completeness of cytoreduction
CT: Computed tomography
HCC: Hepatocellular carcinoma
ICG: Indocyanine green
MRI: Magnetic resonance imaging
PCI: Peritoneal cancer index
PIVKA-II: Protein induced by vitamin K absence or antagonist-II
